# An Atypical Rash in Disseminated Herpes Zoster: A Case Report

**DOI:** 10.7759/cureus.33359

**Published:** 2023-01-04

**Authors:** Tomás Costa, Tiago Gomes, Cândida Fernandes

**Affiliations:** 1 Dermatology, Centro Hospitalar Universitário de Lisboa Central, Lisbon, PRT; 2 Dermatology, Hospital Distrital de Leiria, Leiria, PRT

**Keywords:** herpes zoster virus, maculopapular exanthema, papulovesicular rash, disseminated rash, varicella zoster virus infection

## Abstract

Disseminated cutaneous herpes zoster (DCHZ) is an atypical presentation of herpes zoster (HZ) that mainly affects immunosuppressed patients. Given the potential risk for visceral fatal involvement, prompt recognition of this condition is crucial.

In this case report, we present the case of a 90-year-old male with chronic lymphocytic leukemia under chlorambucil treatment who presented to the emergency department with multiple, converging, crusted papules on his face. He was misdiagnosed with a drug eruption and hospitalized after switching the antibiotic therapy. After one week, the lesions spread in a cephalocaudal pattern, affecting both the trunk and limbs, following which the Dermatology team was consulted. We performed an HZV smear test and initiated acyclovir. Unfortunately, the test was positive, and DCHZ was confirmed. The patient died one week later due to pneumonitis which evolved into a severe acute respiratory distress syndrome.

## Introduction

Herpes zoster (HZ), also known as shingles, is a viral infection caused by the varicella-zoster virus (VZV), a member of the herpesvirus family. VZV is the same virus that causes chickenpox and can be transmitted from person to person through direct contact with the fluid from the blisters or through respiratory secretions. After a person has recovered from chickenpox, VZV remains dormant (inactive) in the body and can reactivate later in life to cause HZ [1].

The risk of developing HZ increases with age, and it is most common in people over the age of 50. It is also more common in people with compromised immune systems, such as those with human immunodeficiency virus (HIV)/acquired immunodeficiency syndrome (AIDS) or cancer, or those taking immunosuppressive medications [2]. The most common symptom of HZ is a painful rash or blisters that typically appear on one side of the face or body along a specific dermatome, with the most common complications including postherpetic neuralgia and ocular compromise [3].

However, in rare cases, especially in people with compromised immune systems, HZ can disseminate and spread to involve multiple dermatomes or other organs [4]. In fact, disseminated cutaneous herpes zoster (DCHZ) is an atypical presentation defined by 20 or more vesicular lesions outside the primary and adjacent dermatomes and can lead to life-threatening complications, such as pneumonia, encephalitis, and hepatitis [5,6]. As such, prompt recognition of DCHZ and initiation of acyclovir treatment are crucial to prevent catastrophic outcomes.

## Case presentation

A 90-year-old male with chronic lymphocytic leukemia (under chlorambucil treatment) presented to the emergency department (ED) with multiple converging and crusted papules on his face. The rash had begun two weeks ago, a few days after he was started on trimethoprim-sulfamethoxazole due to a urinary tract infection. The patient had several comorbidities, including dementia and a transient ischemic attack in the previous year.

When observed in the ED, the patient was conscious and eupneic. The arterial pressure was 169/77 mmHg, and the heart rate was 93 beats/minute. There were no other alterations in the physical examination. The urinary summary test revealed leukocyturia and hematuria. The blood tests revealed an increase in inflammatory biomarkers (leukocytosis and C-reactive protein). A chest X-ray was also performed, but there were no obvious acute changes in the pulmonary parenchyma.

**Figure 1:**
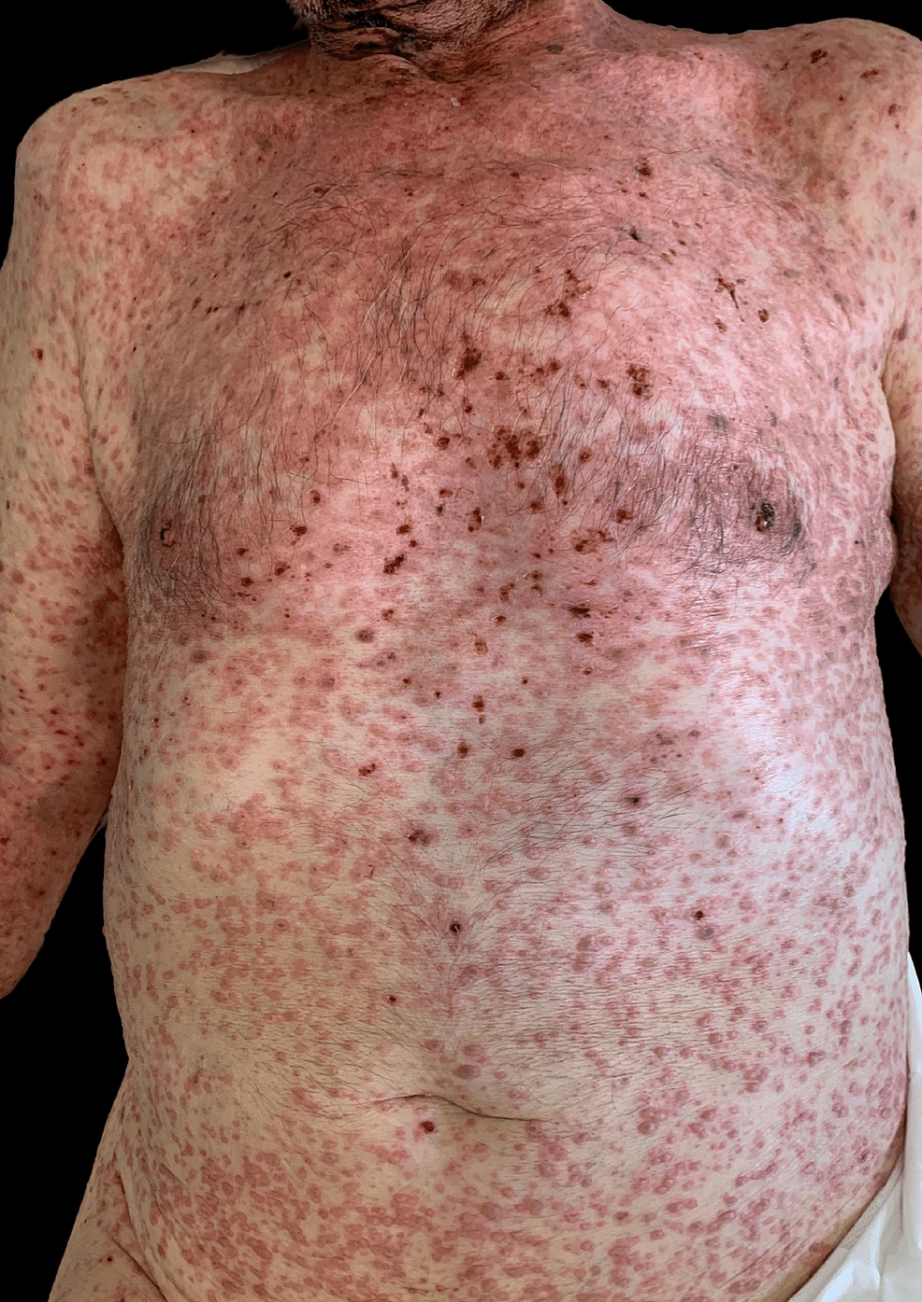
Disseminated papules and vesicles with an erythematous base located on the trunk.

Given the multiple comorbidities and the suspected urinary tract infection, the patient was hospitalized, and piperacillin/tazobactam was initiated. However, after five days of hospitalization, the lesions spread in a cephalocaudal pattern, affecting both the trunk and limbs (Figure 1). The Dermatology team was consulted. On observation, a disseminated dermatosis with multiple vesicles with an erythematous base, suggestive of DCHZ, was noted.

An HZV smear was performed, and acyclovir therapy was promptly initiated. However, on the second day of treatment, the patient presented with respiratory distress, increased secretions, bibasilar crackles on pulmonary auscultation, and periods of oxygen desaturation (SpO_2_ of 87%). A chest radiograph was requested, which revealed bilateral diffuse infiltrates. Blood cultures were negative.

Although the HZV smear confirmed the diagnosis, the patient quickly deteriorated, with long periods of impaired consciousness, increased intercostal retractions, and paradoxical abdominal movement.

Unfortunately, he passed away after two days. Given his age, comorbidities, and a confirmed HZV infection, it was decided not to request an autopsy, and an acute respiratory distress syndrome (ARDS) in the context of his HZV infection was assumed as the cause of death.

## Discussion

DCHZ is an atypical presentation that mainly affects immunocompromised hosts [4-6]. Several mechanisms have been proposed to explain this atypical reactivation pattern in patients with decreased immunity, including impaired T-cell immune response to HZV in patients infected with HIV, or the reduced HZV cellular response and specific immunoglobulin G antibody avidity in transplant solid organ and stem cell recipients [7].

As in the presented case, the differential diagnosis with a drug eruption can be challenging, especially for non-dermatologists. However, it is important to know that every acute vesiculopapular rash with an erythematous base should raise the alert for a zoster infection, especially in patients with an impaired immune system.

If available, the diagnosis can be quickly confirmed by polymerase chain reaction because they are the most sensitive and specific diagnostic tests for detecting varicella DNA in fluid taken from the vesicles [8].

After diagnosing DCHZ, prompt initiation of antiviral therapy is mandatory because it prevents visceral organ involvement and shortens the disease course, thus limiting the chances of chronic sequela. As documented in our patient, the respiratory tract is a frequent site of visceral involvement that is associated with severe complications, such as pneumonia and ARDS [6].

Our case also highlights the benefits of HZV vaccination, especially in older or immunosuppressed patients, in whom there is a higher risk of reactivation. Traditionally limited to live attenuated vaccines, HZV reactivation can now also be prevented by a newly approved recombinant subunit vaccine, HZ/su (Shingrix ®), with a reported efficacy of >90% for confirmed HZ [9]. Given the high morbidity and mortality associated with DCHZ and the recent improvements in vaccination efficacy, HZ prophylaxis is now being widely recommended among immunosuppressed patients [10].

## Conclusions

We reported a case of severe DCHZ in a patient undergoing chemotherapy, initially misdiagnosed as having a drug eruption. This case highlights the importance of considering DCHZ in any immunosuppressed patient presenting with multiple disseminated vesicles with an erythematous base. The patient’s fatal outcome further highlights the importance of a timely diagnosis due to the risk of multisystemic involvement. It also highlights the importance of vaccination against HZV, especially in patients with increased risk for severe and atypical forms of reactivation.
